# Multivariate Quantitative Prediction of Soluble Solids Content, Moisture Content, and Fruit Firmness in ‘Dinosaur Egg’ Apricot Plum via Near-Infrared Spectroscopy with Cross-Parameter Feature Fusion and SHapley Additive exPlanations-Based Optimization

**DOI:** 10.3390/foods14234118

**Published:** 2025-12-01

**Authors:** Yunhai Wang, Zhaoshuai Zhu, Wulan Mao, Kuanbo Cui, Liling Yang, Lina Sun, Wenjie Ma, Wenqiang Ma, Binbin Xiang

**Affiliations:** 1 Agricultural Mechanization Institute, Xinjiang Uygur Autonomous Region Academy of Agricultural Sciences, Urumqi 830091, China; 15689874356@163.com (Y.W.); zzsxj11@163.com (Z.Z.); maowulan@xaas.ac.cn (W.M.); widewave@126.com (K.C.); yangliling627@163.com (L.Y.); lina_sun@xaas.ac.cn (L.S.); mwenjie729@163.com (W.M.); 2 College of Mechanical Engineering, Xinjiang University, Urumqi 830046, China

**Keywords:** apricot plum, cross-parameter feature fusion, near-infrared spectroscopy, soluble solids content, moisture content, fruit firmness

## Abstract

To meet market demand for fresh ‘Dinosaur Egg’ Apricot plum and realize effective quality classification, this study developed a non-destructive quality evaluation method using near-infrared spectroscopy (NIRS) with cross-parameter feature fusion. Spectral data were preprocessed, and key bands were screened via Competitive Adaptive Reweighted Sampling (CARS) and Shuffled Frog Leaping Algorithm (SFLA). Partial Least Squares Regression (PLSR) models for soluble solids content (SSC), moisture content (MC), and fruit firmness (FF) were established. Chemical index features were fused with FF-related preliminary features, and SHapley Additive exPlanations (SHAP) optimized feature contribution. Final models showed high performance: SSC ($R_{c}^{2}$ = 0.9354, $R_{p}^{2}$ = 0.9302, RMSE = 0.5212° Brix), MC ($R_{c}^{2}$ = 0.9367, $R_{p}^{2}$ = 0.9314, RMSE = 5.037 × 10^−5^), and FF ($R_{c}^{2}$ = 0.8151, $R_{p}^{2}$ = 0.7986, RMSE = 1.2710 N). This strategy improved the multi-quality detection accuracy, especially for FF, and provides technical support for intelligent fruit grading.

## 1. Introduction

Against the backdrop of sustainable global agriculture and the growing demand for food quality and safety, the accurate evaluation of characteristic fruits has become a key requirement for upgrading the industrial chain [1]. *Apricot plum*, as an excellent variety bred by intergeneric hybridization between apricot (*Prunus armeniaca*) and plum (*Prunus salicina*) [2], is rich in vitamins, minerals, and antioxidants [3]. In recent years, it has become an important economic crop for promoting the development of regional characteristic agricultural economies [4]. Quality indicators such as the soluble solids content, moisture content, and firmness of the fruit are not only an important basis for consumer choice [5], but also a key parameter for guiding planting optimization, determining harvest time, optimizing storage strategies, and post-harvest grading. Therefore, the efficient detection of these indicators is of great practical significance for the high-quality development of the *Apricot plum* industry [6].

Traditional quality detection depends on physical and chemical analysis methods [7], such as calculating sugar content using a refractometer and measuring hardness using a texture analyzer. Although these methods provide a high accuracy, they require destructive sampling and involve complex operation, a long analysis time, and a high cost, which are difficult to attain when meeting the needs of large-scale and real-time quality monitoring in modern agricultural production [8]. Moreover, techniques like chromatography (e.g., GC-MS, HPLC) and spectroscopy (e.g., Raman spectroscopy) have been widely applied in food analysis [9,10]. Despite their precision, their high cost and operational complexity restrict their use for rapid field-based fruit quality evaluation.

As a non-destructive detection technology, near-infrared spectroscopy (NIRS) has been widely used in the field of agricultural product quality detection due to its advantages of speed, non-destructive nature, multi-index synchronous analysis, and environmental friendliness [11]. NIRS has shown good application prospects in the prediction of fruit sugar content, acidity, hardness, and other indicators [12,13]. However, most existing studies focus on the modeling and prediction of a single quality attribute and typically use only the spectral features associated with that attribute [12,14,15,16]. This single-dimensional modeling approach ignores the physiological relationships among fruit qualities, such as the synergistic regulation mechanism of soluble solids accumulation and water content change [17], or the interaction between firmness degradation and sugar accumulation during fruit development [18]. As a result, useful spectral information may remain underutilized, limiting model accuracy and robustness.

In recent years, multi-source information fusion modeling has become an important direction to improve near-infrared prediction performance [19]. By integrating features associated with different qualities, the model ‘s ability to capture complex quality changes can be enhanced [20]. At the same time, near-infrared spectral data typically contain a large number of redundant bands and noise, and the scientific screening of characteristic bands is the core step for optimizing the performance of the model [21]. The SHapley Additive exPlanations (SHAP) method, based on the Shapley value principle in game theory, provides an interpretable and quantitative approach to evaluating feature importance and improving model simplicity and generalization [22]. Although the fusion modeling and SHAP interpretation methods have been applied in the quality detection of agricultural products [23], there is still a lack of research on the multi-quality collaborative prediction of *Apricot plum*. How to construct a fusion model through quality correlation analysis and combine SHAP to optimize feature selection has become a key issue in improving the NIRS prediction accuracy of *Apricot plum* quality.

Given these gaps, the primary objectives of this study are as follows: (1) To combine different feature extraction methods and modeling approaches to develop predictive models for three key quality indicators of *Apricot plum*: soluble solids content (SSC), moisture content (MC), and fruit firmness (FF). (2) To explore the possibility of multi-quality fusion modeling by analyzing the correlations between chemical and physical indicators, and use the SHAP method to optimize feature selection, thereby improving the accuracy of *Apricot plum* quality prediction models.

This study will provide valuable insights into the multi-quality optimization of *Apricot plum* using NIRS and offer a more robust framework for real-time, large-scale quality monitoring in the fruit industry.

## 2. Materials and Methods

### 2.1. Sample Preparation

**Figure 1:**
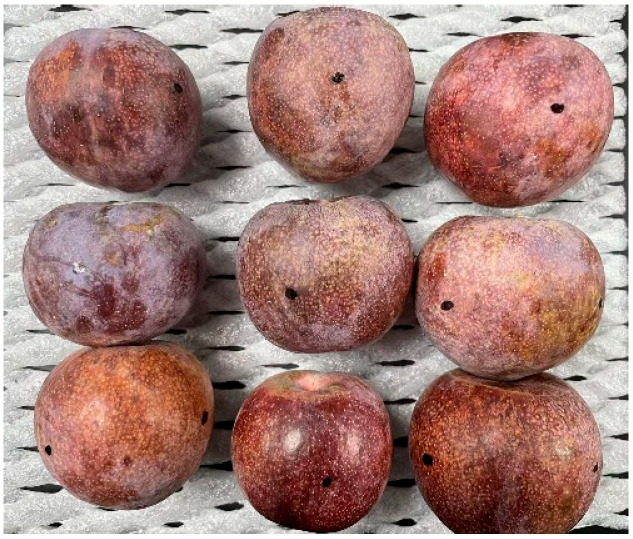
*Apricot plum* samples showing the three marked equatorial points for spectral acquisition, avoiding stem and pedicel regions.

Fresh ‘Dinosaur Egg’ *Apricot plums* (*n* = 120) were harvested from Jiuding Market (Urumqi, China) in July 2024 at commercial maturity. Samples were selected for their uniform size (diameter: 6.5 ± 0.5 cm) and an absence of defects. After surface cleaning, fruits were equilibrated at 25 °C for 12 h. Three equatorial points per fruit, spaced at 120° as sampling points, were marked for spectral acquisition, ensuring that the spectral measurements avoided the stem and pedicel regions, which are known to cause signal heterogeneity [24], as shown in Figure 1.

### 2.2. Spectral Information Collection

**Figure 2:**
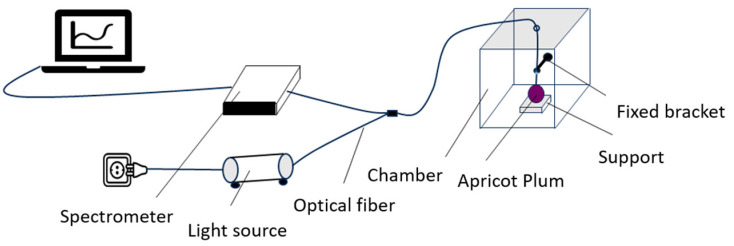
Near-infrared spectral information acquisition schematic diagram.

Near-infrared spectroscopy (NIRS) is a spectroscopic analytical technique based on molecular vibrations and energy level transitions, commonly used to detect components such as moisture, sugar content, and acidity in substances (Figure 2). In the near-infrared wavelength range (700–2500 nm), the vibrational overtones and combination bands of C–H, O–H, and N–H bonds produce characteristic absorptions that reflect the chemical composition and physical properties of a sample. Compared with traditional physicochemical analytical methods (such as refractometers, texture analyzers, or chromatography), NIRS offers advantages including being non-destructive, rapid, capable of simultaneous multi-parameter analysis, and environmentally friendly. It enables large-scale, real-time quality detection without damaging the sample. Therefore, this study employs NIRS for quality analysis of *Apricot plum* fruits, aiming to efficiently predict and comprehensively evaluate key indicators such as sugar content, moisture, and firmness.

The near-infrared diffuse transmission measurement protocol adopted in this study is based on acquiring transmitted light spectra after near-infrared radiation passes through the internal structure of the sample and is received by the detector. During measurement, the *Apricot plum* sample is placed in the transparent region of a specialized test holder, ensuring that the fruit surface is intact, clean, and closely fitted to the holder to minimize light scattering interference. The sample is placed on a test support, and the optical fiber (QR400-7-VIS-NIR, Ocean Insight, Orlando, FL, USA) position is fixed to ensure that the optical fiber probe is attached to the sample surface and connected to a 20 W halogen light source (HL-2000-HP, Ocean Insight, Orlando, FL, USA). Flame-NIR + fiber spectrometer (Ocean Insight, Orlando, FL, USA) was used for spectral acquisition. The wavelengths of transmittance range from 970 to 1700 nm with a 5.6 nm increment, the number of spectral acquisitions for each marker point is 3 times, and the average value is taken as the output spectrum of a single marker point [25,26,27,28]. After obtaining the output spectra of the three marker points of each sample, the average value is taken as the spectral information collected by a sample to ensure the accuracy of the collected spectral signals [29,30]. Each sample spectrum has 129 wavelength points. Since the head and tail wavelength regions usually contain high noise and instrument background signals, 15 wavelength points are deleted from the head and tail wavelength regions, and the final spectrum of each sample has 99 wavelength points [31].

### 2.3. Quality Index Determination

#### 2.3.1. Soluble Solids Content

Soluble solids content (SSC) is defined as the total concentration of sugars and other soluble compounds within the fruit, serving as a key indicator of its sweetness and overall quality. To analyze the SSC value, the fruit pulp was carefully excised and pressed to extract the juice. About 3–5 mL of juice is obtained per press, with approximately 8 g of fruit pulp required to obtain this volume of juice. In order to ensure the representativeness of the data, the juice extraction of each fruit was repeated twice, each time from a different pulp area. The juice was then analyzed using a digital refractometer (PAL-1, ATAGO Co., Ltd., Tokyo, Japan), and the measured value was reported in degrees Brix (°Brix). After that, the two results are averaged.

#### 2.3.2. Moisture Content

For moisture content (MC), fresh samples were taken and the fruit was cut into small pieces with a blade to ensure uniform samples. A certain amount of fresh weight sample (about 5 g) was weighed and then placed in a hot air oven pre-heated to 105 ± 1 °C (DHG-9240A, Sanqing Instrument Co., Ltd., Suzhou, Jiangsu, China) for drying. During the drying process, the sample is continuously heated until the quality is stable, that is, the weight difference between two consecutive weighing is less than 0.01 g, indicating that the sample has reached a constant weight.

#### 2.3.3. Fruit Firmness

The firmness was measured by a fruit firmness (FF) tester (GY-4, BOTE Instrument Co., Ltd., Lianyungang, China) with a measurement accuracy of ±0.5%. In this study, two symmetrical points on the equator line of each *Apricot plum* sample were used as the hardness acquisition positions. A stainless-steel cylindrical probe with a diameter of 6 mm was used to penetrate the meat at a depth of 1 cm at a speed of 1 mm/s. The maximum force required for the probe to penetrate the fruit sample was used to calculate the hardness. Taking N as a unit, the average value of two sampling points was taken as the fruit hardness value of each sample, so as to reduce the influence of different fruit parts and improve the accuracy of measurement [32].

### 2.4. Analysis of Relationship

In this study, Pearson’s correlation coefficient was used to analyze the correlation between FF and SSC and the correlation between FF and MC. Correlation analysis is a statistical method used to measure the strength and direction of the linear relationship between variables. In the study of fruit physical and chemical parameters, correlation analysis can not only identify the potential relationship between indicators, but also help to screen out the parameters that have the greatest impact on fruit quality, and provide a scientific basis for subsequent analysis and strategy formulation.

Firstly, three physical and chemical indexes (such as SSC, MC, and FF) of 120 samples of *Apricot plum* were collected, and the mean and standard deviation of each parameter were calculated. Then, the Pearson’s correlation coefficient is used to analyze the linear correlation between the parameters [33,34].

### 2.5. Data Preprocessing and Regression Modeling Methods

In order to suppress the influence of baseline drift and random noise in the spectral signal, MatlabR2024a (MathWorks, Natick, MA, USA) was used to process the data, and the spectral data were preprocessed by Min–Max Normalization, Multiplicative Scatter Correction (MSC), and Standard Normal Variate (SNV) [35,36,37,38].

When establishing the quality prediction model of *Apricot plum*, some bands contain strong noise and interference, and inputting the full spectral band into the model will lead to a long calculation time and low prediction accuracy. In order to reduce the complexity of data and improve the correlation of data, it is necessary to extract the characteristic bands. Competitive Adaptive Reweighted Sampling (CARS) [39,40] and Shuffled Frog Leaping Algorithm (RFLA) [41] were used to extract the feature bands, and the selected effective bands were utilized as model inputs.

### 2.6. Regression Modeling Methods

After preprocessing the spectral data, the regression prediction models of SSC, MC, and FF were constructed using the Least Squares Regression (PLSR) [42,43] method. The maximum number of PLS principal components used in this study was set to 20. The Kennard–Stone algorithm was used to divide the dataset into a training set (80 samples) and a validation set (40 samples), ensuring uniform coverage of the spectral space. Training set R-squared ($R_{c}^{2}$), test set R-squared ($R_{p}^{2}$), and Root Mean Square Z-value (RMSE) were used to evaluate the performance of the model [44,45].

### 2.7. Model Interpretability

In order to further improve the accuracy of model prediction and analyze the correlation between the three quality indexes of *Apricot plum*, SHapley Additive exPlanations (SHAP) was used to fuse and screen the characteristic bands in different quality prediction models.

SHAP is an interpretable machine learning framework based on game theory, which explains how each feature contributes to the prediction output of a model. Specifically, SHAP calculates the marginal contribution of every feature by considering all possible combinations of features. This process ensures that the contribution of each variable is fairly allocated according to its influence on the model output, improving model transparency and interpretability [46,47,48]. In simple terms, SHAP values quantify how much each spectral band increases or decreases the predicted value relative to the model’s average output. Positive SHAP values indicate that a feature increases the prediction result, while negative values indicate the opposite.

SHAP satisfies the additive principle, which means that the sum of the importance scores of all features is equal to the output of the model, and its calculation formula is shown in Formula (1).

(1) $$\phi_{i}=\underset{S\subseteq N\backslash \left\{i\right\}}{\sum }\frac{\left|S\right|!\left(\left|N\right|-\left|S\right|-1\right)!}{\left|N\right|!}\left(f\cup \left(S\left\{i\right\}\right)-f\left(S\right)\right)$$

where $\phi_{i}$ denotes the SHAP value of the characteristic wavelength i in the FF prediction model, N denotes the total number of spectral characteristic wavelengths used in the modeling process, S denotes any subset of the spectral characteristic wavelength set excluding wavelength i, $f\left(S\right)$ denotes the model prediction result using only the wavelength subset, |S| denotes the number of wavelengths contained in subset S, and |N| denotes the total number of all spectral characteristic wavelengths used in the modeling process.

## 3. Results

### 3.1. Spectral Data Preprocessing

**Figure 3:**
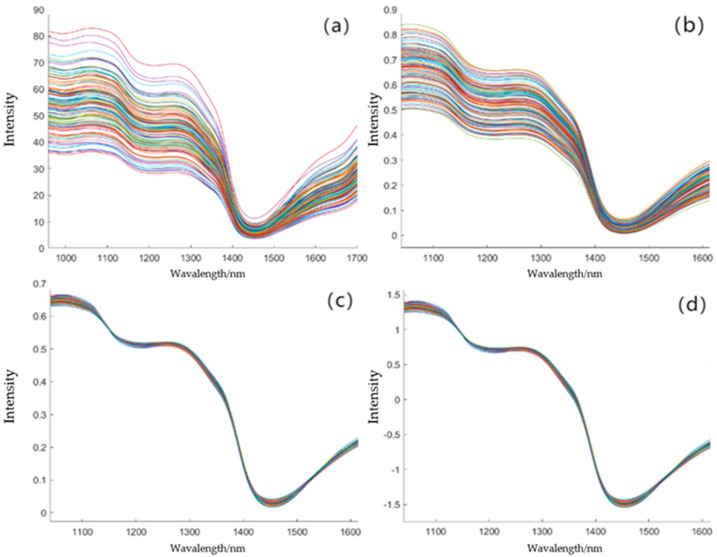
Under different pretreatment methods, the near-infrared spectral curves of *Apricot plum*: (**a**): original spectral curve; (**b**): Min–Max Normalization and SG; (**c**): Min–Max Normalization and MSC; (**d**): Min–Max Normalization and SNV.

Figure 3 shows the original spectral curve of the *Apricot plum* sample, the spectral curve after Min–Max Normalization and SG pretreatment, the spectral curve after Min–Max Normalization and MSC pretreatment, and the spectral curve after Min–Max Normalization and SNV pretreatment. The wavelength range of the original spectral curve is 970–1700 nm.

From Figure 3a, it can be seen that the spectra of all *Apricot plum* samples show the same change trend, with troughs at 1180 nm and 1445 nm and peaks at 1285 nm. The band near 1180 nm may be related to the change in cell wall components (such as cellulose) and affect fruit firmness [49,50]. The characteristic absorption peaks observed at 1445 nm correspond to O-H stretching vibrations in water molecules [11,51], while the 1285 nm peak reflects C-H combinations in soluble sugars [12]. The noise beyond 1625 nm aligns with instrument-dependent thermal drift, justifying our 1040–1625 nm range selection. Because the spectrum is affected by baseline drift before the wavelength of 1040 nm and after the wavelength of 1625 nm, this drift makes the whole curve of the spectrum unable to accurately reflect the true characteristics of the measured sample. Therefore, the spectral data with a wavelength range of 1040–1625 nm are selected as the target spectral data. There are 99 characteristic bands in the selected range. From the original spectral curve, it can be seen that the spectrum has considerable scattering and noise. Therefore, the spectral data in the selected wavelength range of 1040–1625 nm are preprocessed. Figure 3b is the spectral curve after Min–Max Normalization and SG pretreatment. It can be seen that the trend of the spectral curves presented by each sample is consistent, but the spectrum still has considerable scattering characteristics. Figure 3c is the spectral curve after Min–Max Normalization and MSC pretreatment. It effectively corrects the scattering of the spectrum and adjusts the system differences between different samples. In Figure 3d, the spectral curve after Min–Max Normalization and SNV preprocessing not only has a good correction effect on the scattering effect, but also ensures that the characteristics of each sample have a more consistent scale, which can effectively reduce the error caused by the change in spectral amplitude.

### 3.2. Apricot Plum Quality Modeling

**Figure 4:**
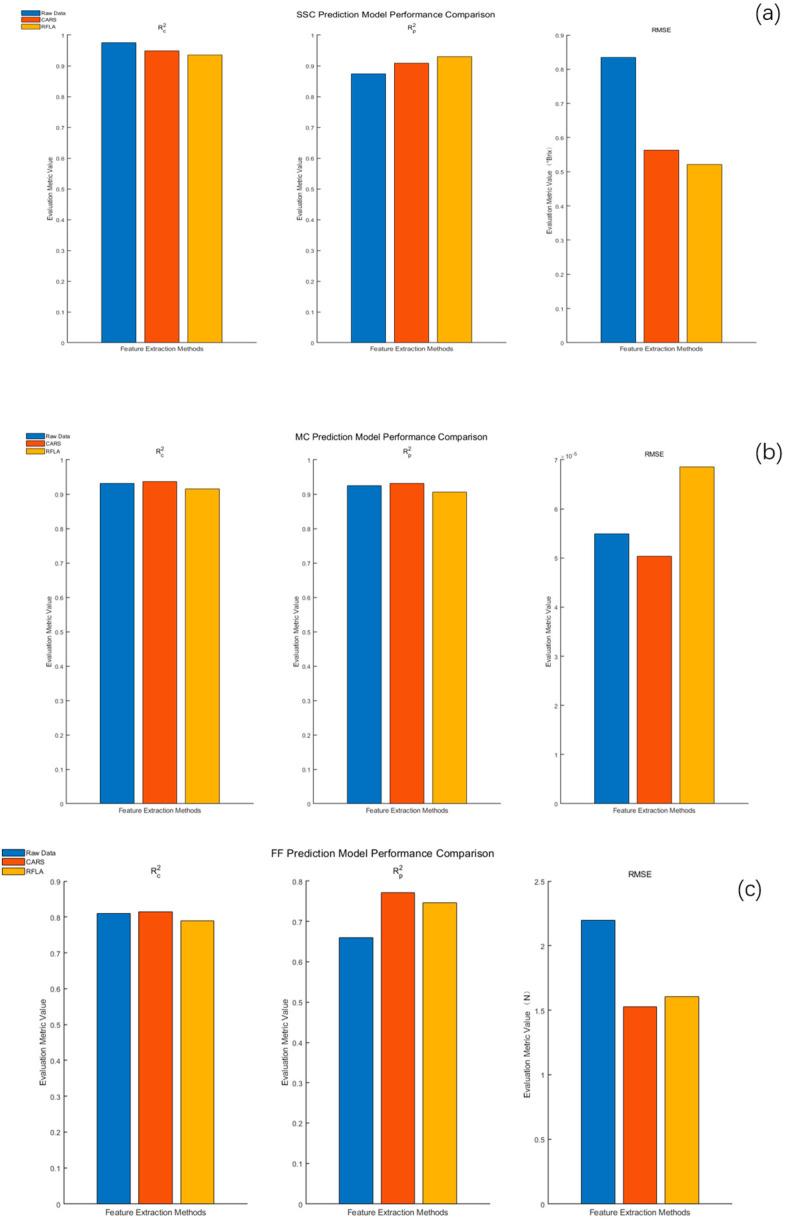
Comparative analysis of PLSR model prediction performance of *Apricot plum* quality parameters based on different feature extraction methods: (**a**): SSC PLSR model comparison; (**b**): MC PLSR model comparison; (**c**): FF PLSR model comparison.

The prediction models of SSC, MC, and FF were established by using the characteristic band extraction methods (CARS and RFLA) and regression modeling method (PLSR). The results are shown in Figure 4.

It can be seen from Figure 4a that the PLSR-based method showed good prediction performance in the established prediction model of the SSC of *Apricot plum*. Specifically, the RFLA and PLSR combined model obtained a high degree of fitting on the training set ($R_{c}^{2}$ = 0.9354), and also showed excellent generalization ability on the prediction set ($R_{p}^{2}$ = 0.9302, RMSE = 0.5212° Brix), showing significantly better model stability than other methods. It can be seen from Figure 4b that in the established prediction model of the MC of *Apricot plum*, the PLSR model still maintained a robust performance after feature extraction. The CARS and PLSR combination achieved a higher prediction accuracy while simplifying the data dimension ($R_{p}^{2}$ = 0.903, RMSE = 1.12), which was better than the full-spectrum PLSR ($R_{p}^{2}$ = 0.901, RMSE = 1.14). It can be seen from Figure 4c that in the established prediction model of *Apricot plum* fruit firmness, the performance of all models did not reach the ideal level. Although the model combining CARS and PLSR showed a better performance than other methods, its $R_{c}^{2}$, $R_{p}^{2}$, and RMSE were 0.8138, 0.7715, and 1.5280 N, respectively, but the feature expression still needed to be further optimized.

Based on the modeling results shown in Figure 4a,b, RFLA-PLSR and CARS-PLSR performed well in SSC and MC prediction, respectively, reflecting the good synergistic effect between PLSR and feature extraction methods. This result establishes a methodological principle for the optimization of the prediction model to be carried out and points out the importance of further research in the field of fruit firmness prediction.

### 3.3. Optimization of Fruit Firmness Prediction Model

In order to further explore the relationship between the three variables of SSC, MC, and FF, and to reveal the interaction between quality indicators, a correlation analysis of these three variables was carried out.

Table 1 shows the results of the correlation analysis (Pearson’s correlation coefficient) of the SSC, MC, and FF of *Apricot plum*. It can be seen from Table 1 that the r value of FF and SSC is 0.81, and the r value of MC is −0.79. This showed that the FF of *Apricot plum* had a marked positive correlation with its SSC and a strong negative correlation with its MC.

In order to further improve the FF prediction accuracy of *Apricot plum*, the characteristic bands of the three quality prediction models were combined. Table 2 shows the characteristic bands in the three quality prediction models. From Table 2, it can be seen that the establishment of the three models used 20, 9, and 20 characteristic bands. The bands used in the three quality prediction models were screened and combined. After removing the repeated bands, the combined 38 bands were used to re-optimize the model. Although the original spectra show significant absorption characteristics at 1180 nm, 1285 nm, and 1445 nm (Figure 3a), the final modeling bands (such as 1313 nm of SSC model and 1425 nm of MC model) are different from them. This may be due to the fact that the SNV preprocessing weakens the original peak-to-valley contrast, so that the effective signal of the weak feature region (such as 1100–1200 nm) is highlighted, and the feature selection algorithm preferentially retains the band with a high statistical correlation with the target variable (such as 1075 nm of the FF model) [52].

**Figure 5:**
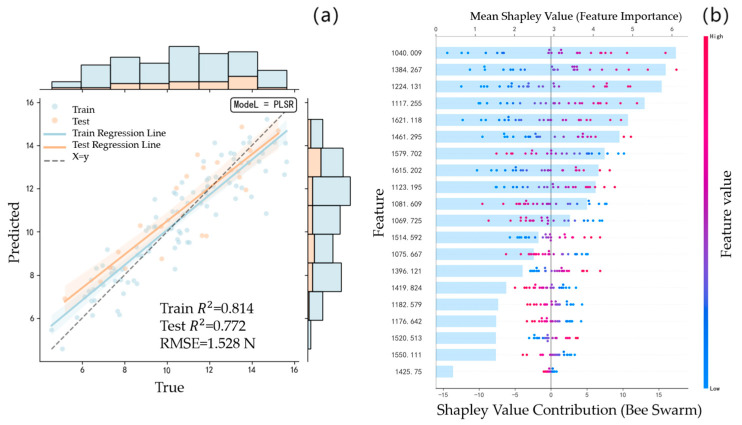
The FF model of *Apricot plum* without fusion of characteristic wavelength: (**a**): PLSR model prediction results; (**b**): SHAP value distribution of each characteristic wavelength in the model.

**Figure 6:**
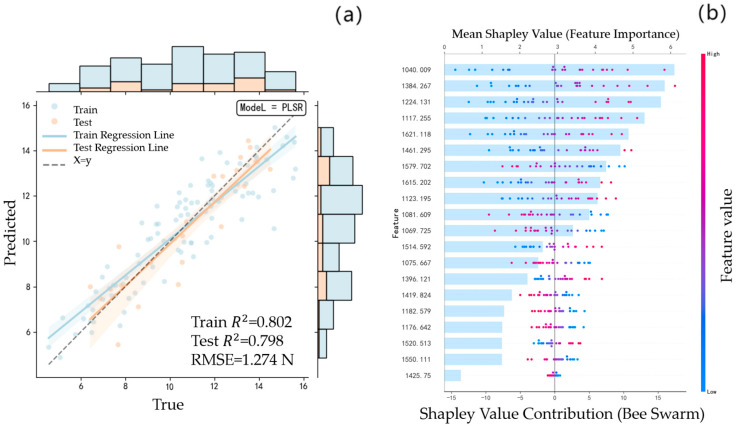
The FF model of *Apricot plum* fused with characteristic bands: (**a**): PLSR model prediction results; (**b**): SHAP value distribution map of each band in the model.

The same modeling method is used to re-establish the FF prediction model for the spectral data of the unfused feature band and the spectral data of the fused feature band. Figure 5a is the PLSR model prediction result of the unfused feature bands, and Figure 6a is the PLSR model prediction result of the fused feature bands. Compared with the modeling results without fusion, $R_{c}^{2}$ decreased from 0.814 to 0.802, whereas $R_{p}^{2}$ and RMSE improved from 0.772 and 1.528 N to 0.798 and 1.274 N, respectively. This indicates that the prediction accuracy of the initial fused feature band has demonstrated a measurable improvement.

The *Apricot plum*’s FF prediction model was constructed using the feature band fusion strategy, and the feature bands were increased from 20 to 38 of the benchmark model, which effectively improved the data abundance but simultaneously increased the model complexity. In order to balance the accuracy and complexity of the model, the SHAP value is used to optimize the contribution of the characteristic band by multi-stage screening. As shown in Figure 5b and Figure 6b, the distribution of SHAP values demonstrates that the fusion model enhances the interaction effects among features because of the increased number of spectral bands. In addition, several bands that originally exhibited low contributions become significantly more important after fusion, indicating an improved representation of relevant spectral information. Table 3 shows the model comparison results of the screening and optimization of SHAP values in different stages under the two conditions. The results in Table 3 show that the evaluation indexes of the benchmark model are significantly degraded after the second screening ($R_{c}^{2}$ decreased from 0.8117 to 0.7816, $R_{p}^{2}$ decreased from 0.7620 to 0.6918, and RMSE increased from 1.592 N to 2.062 N), and there is considerable under-fitting after the third screening ($R_{c}^{2}$ = 0.4432, $R_{p}^{2}$ = 0.5365), which confirms that the excessive deletion of features leads to the loss of key information. In contrast, the number of bands of the fusion model decreased from 38 to 17 after two screenings, and still maintained a high prediction accuracy ($R_{c}^{2}$ = 0.8151, $R_{p}^{2}$ = 0.7986, RMSE = 1.271 N), achieving the optimal balance between complexity and generalization ability. It is worth noting that when the number of screenings increases to four times, the fusion model also presents an under-fitting phenomenon, highlighting the criticality of the feature screening threshold. The results show that the second-stage screening strategy based on the SHAP value reduces the number of characteristic wavelengths by 55.26% while maintaining the original prediction accuracy of the fusion band model, which provides an effective technical path for spectral data feature optimization.

## 4. Discussion

In this study, a prediction method for *Apricot plum* quality based on near-infrared spectroscopy and cross-parameter feature fusion was successfully established. This method overcomes the limitations of traditional destructive detection, does not require sample preparation, avoids fruit loss, and can continuously and dynamically monitor the same fruit (such as tracking hardness changes during storage), providing technical support for the standardization of breeding screening and post-harvest grading.

Near-infrared spectroscopy has formed a large-scale application ecosystem in the agricultural field due to its fast and non-destructive advantages [53,54]. In the prediction of stone fruit quality, near-infrared technology has shown high-precision potential [25,55,56]. These applications provide a methodological basis for this study and highlight the need to improve the accuracy of fruit quality prediction. Compared with the SSC prediction results of three varieties of plum ($R_{p}^{2}$ = 0.94) by Louw & Theron [15], through the established multi-variety prediction model, Amoriello et al. [57] used a multi-layer perceptron artificial neural network to predict the DM of apricot ($R_{p}^{2}$ = 0.86). In this study, CARS and SFLA were used to screen the characteristic bands, and the SSC and MC prediction models constructed by PLSR were combined to achieve the accuracy of the model ($R_{p}^{2}$ > 0.93), which verified the high reliability of near-infrared spectroscopy in the quantitative analysis of Rosaceae fruits. For the prediction of fruit firmness, compared with the traditional single-parameter model, this study introduces the SHAP interpretability framework. By quantifying the positive contribution of SSC-related bands to FF (SHAP > 0) and the negative influence of MC-related bands (SHAP < 0), the physiological coupling mechanism of sugar accumulation strengthening the cell wall and water loss weakening tissue was revealed. The prediction accuracy of FF was improved to $R_{p}^{2}$ = 0.7986, RMSE = 1.271 N, which was better than that of the single feature extraction method (such as $R_{p}^{2}$ = 0.7715 of the CARS-PLSR model). Compared with the best accuracy ($R_{p}^{2}$ = 0.72, RMSE = 3.96 N) obtained by Puneet Mishra et al. [58] with a PLSR model for avocado under two different relative humidity conditions, the performance of the model has been improved.

The SHAP-CARS-PLSR model system constructed in this study has certain industrial application potential. The band dimension is compressed by 55.26% through feature contribution screening, which meets the real-time computing requirements of embedded devices. At the same time, it breaks through the limitations of the traditional ‘black box model’, realizes the interpretable cross-parameter correlation analysis, and provides the mechanism basis for quality control. Aldrees et al. [46] emphasized ‘the quantitative value of SHAP to multi-feature interaction’ in water quality prediction.

Future research needs to focus on breaking through three limitations: in terms of sample collection, the sample size of this study only covers a single harvest season, and future research needs to include samples under multi-season and multi-storage conditions to verify the robustness of the model; considering the hardware, the spectral range should be extended to 2500 nm to capture the cellulose characteristic peak at 2100 nm, which makes up for the lack of current hardness characterization. Algorithmically, the model could be combined with the One-Dimensional Convolutional Neural Network deep learning framework to optimize nonlinear feature interaction modeling. The results of this study not only provide a core tool for the post-harvest intelligent grading of *Apricot plum*, but also establish a reusable methodological framework for multi-quality testing of stone fruit crops. Although direct comparison with classical analytical methods was not conducted in this study, similar spectral characteristics and prediction trends have been reported in previous works [59,60,61], which confirms the feasibility and reliability of the proposed approach.

## 5. Conclusions

This study proves that the modeling theory of NIRS combined with cross-parameter fusion can effectively realize the detection of multiple quality indicators of *Apricot plum*. According to the characteristics of different quality indexes, the modeling path was selected, and the prediction models of SSC, MC, and FF of *Apricot plum* were established. A multi-dimensional fusion detection framework of spectral characteristics–physical parameters correlation was proposed, and an integrated model system for predicting multi-quality indicators was established, which provided a theoretical model for the rapid and non-destructive detection of fruit post-harvest quality. In the process of optimizing the fruit firmness prediction model, the feature dimension of the fruit firmness prediction model is reduced by the SHAP band optimization mechanism, which ensures the prediction accuracy and significantly reduces the model complexity. This method provides theoretical support for the rapid grading of *Apricot plum* after delivery.

## Figures and Tables

**Table 1:** The correlation analysis of three quality parameters. SSC (° Brix); MC (%); FF (N).

Quality Index	SSC	MC	FF
SSC	1	−0.96	0.81
MC	−0.96	1	−0.79
FF	0.81	−0.79	1

**Table 2:** The characteristic bands used to establish the best model for each of the three qualities based on the method of establishing the optimal model and the obtained spectral band (nm).

Quality Type	Modeling Method	Spectral Waveband (nm)
SSC	RFLA-PLSR	1200.389; 1206.325;1265.668; 1313.122; 1319.052; 1324.982; 1330.912; 1384.267; 1419.824; 1425.75; 1431.675; 1520.513; 1526.433; 1532.353; 1567.867; 1573.785; 1579.702; 1585.62; 1591.537; 1621.118
MC	CARS-PLSR	1069.725; 1188.516; 1230.065; 1265.668; 1313.122; 1354.628; 1425.75; 1538.273; 1573.785
FF	CARS-PLSR	1040.009; 1069.725; 1075.667; 1081.609; 1117.255; 1123.195; 1176.642; 1182.579; 1224;131; 1384.267; 1396.121; 1419.824; 1425.75; 1461.295; 1514.592; 1520.513; 1550.111; 1579.702; 1615.202; 1621.118

**Table 3:** Comparison of model prediction performance based on SHAP-selected feature bands under different significance thresholds.

Range	Data Type	$R_{c}^{2}$	$R_{p}^{2}$	RMSE (N)
Incipient	No Fusion (20)	0.8138	0.7715	1.528
	Fusion (38)	0.8017	0.7981	1.274
P<2	No Fusion (14)	0.8117	0.7620	1.592
	Fusion (25)	0.8272	0.7958	1.288
P<4	No Fusion (10)	0.7816	0.6918	2.062
	Fusion (17)	0.8151	0.7986	1.271
P<6	No Fusion (5)	0.4432	0.5365	3.100
	Fusion (15)	0.8277	0.7769	1.408
P<8	No Fusion (-)	-	-	-
	Fusion (12)	0.8063	0.8336	1.050

Note: *P* denotes the SHAP value. The number in parentheses after each data type indicates the number of feature bands used for modeling after filtering.

## Data Availability

The data presented in this study are available on request from the corresponding author. The data are not publicly available due to privacy restrictions.
